# The CAT Scan

**DOI:** 10.3201/eid1404.071651

**Published:** 2008-04

**Authors:** Ronald O. Valdiserri

I enter your portal through a scrim of invisible rays,

beads of energy outnumbering my regrets.

Supine on a cold bed, part supplicant, part sacrifice.

Like all captives, fearing judgment.

***Woosh,*** then comes the warm tingle of the dye,

everywhere at once.

It feels like a cleansing…

washing away debris, debt, equivocation.

*“no,”* silently, I correct myself,

acknowledging the iodine’s more melancholy assignment:

building a luminous marquee around 56 years of imperfection and wear.

And the lights blink yellow.

